# The Mitochondrial Hsp90 TRAP1 and Alzheimer’s Disease

**DOI:** 10.3389/fmolb.2021.697913

**Published:** 2021-06-18

**Authors:** Françoise A. Dekker, Stefan G. D. Rüdiger

**Affiliations:** ^1^ Medicinal Chemistry, Amsterdam Institute of Molecular and Life Sciences (AIMMS), Vrije Universiteit Amsterdam, Amsterdam, Netherlands; ^2^ Cellular Protein Chemistry, Bijvoet Center for Biomolecular Research, Utrecht University, Utrecht, Netherlands; ^3^ Science for Life, Utrecht University, Utrecht, Netherlands

**Keywords:** protein folding, proteostasis, molecular chaperones, protein quality control, protein aggregation, neurodegeneration, mitochondria

## Abstract

Alzheimer’s Disease (AD) is the most common form of dementia, characterised by intra- and extracellular protein aggregation. In AD, the cellular protein quality control (PQC) system is derailed and fails to prevent the formation of these aggregates. Especially the mitochondrial paralogue of the conserved Hsp90 chaperone class, tumour necrosis factor receptor-associated protein 1 (TRAP1), is strongly downregulated in AD, more than other major PQC factors. Here, we review molecular mechanism and cellular function of TRAP1 and subsequently discuss possible links to AD. TRAP1 is an interesting paradigm for the Hsp90 family, as it chaperones proteins with vital cellular function, despite not being regulated by any of the co-chaperones that drive its cytosolic paralogues. TRAP1 encloses late folding intermediates in a non-active state. Thereby, it is involved in the assembly of the electron transport chain, and it favours the switch from oxidative phosphorylation to glycolysis. Another key function is that it ensures mitochondrial integrity by regulating the mitochondrial pore opening through Cyclophilin D. While it is still unclear whether TRAP1 itself is a driver or a passenger in AD, it might be a guide to identify key factors initiating neurodegeneration.

## Introduction

Alzheimer’s Disease (AD) is characterised by progressive cognitive decline (Goedert and Spillantini, 2006; Crews and Masliah, 2010; Weller and Budson, 2018). While widely investigated, there is still no cure available, only treatments providing symptomatic relief (Yiannopoulou and Papageorgiou, 2013; Canter et al., 2016; Long and Holtzman, 2019). Age is the single most risk enlarging factor, increasing prevalence of the disease with the increasing age of citizens (Hou et al., 2019). Major hallmarks of AD are two protein aggregates found in the brain; β-amyloid (Aβ) forming extracellular senile plaques (SPs) and Tau proteins forming neurofibrillary tangles (NFTs) inside the cell (Kidd 1963; Ittner and Götz, 2011; Panza et al., 2019).

The formation of aggregates, such as NFTs and SPs in AD, constitutes a malfunctioning of the cellular Protein Quality Control (PQC) (Miller et al., 2015; Mok et al., 2018). This system can help the (re)folding of proteins, avoid the formation of aggregates, aid in translocating proteins, and degrade proteins when they are beyond repair (Finley, 2009; Koren III et al., 2009; Graham et al., 2017). PQC capacity decreases during aging (Brehme et al., 2014; Hipp et al., 2019). This decline further amplifies in the brains of AD’s patients (Brehme et al., 2014; Xu et al., 2019; Koopman and Rüdiger, 2020). Therefore, the PQC system is a potential target for novel therapeutic strategies for neurodegenerative diseases caused by protein aggregation such as Alzheimer’s Disease (Blair et al., 2014).

**FIGURE 1:**
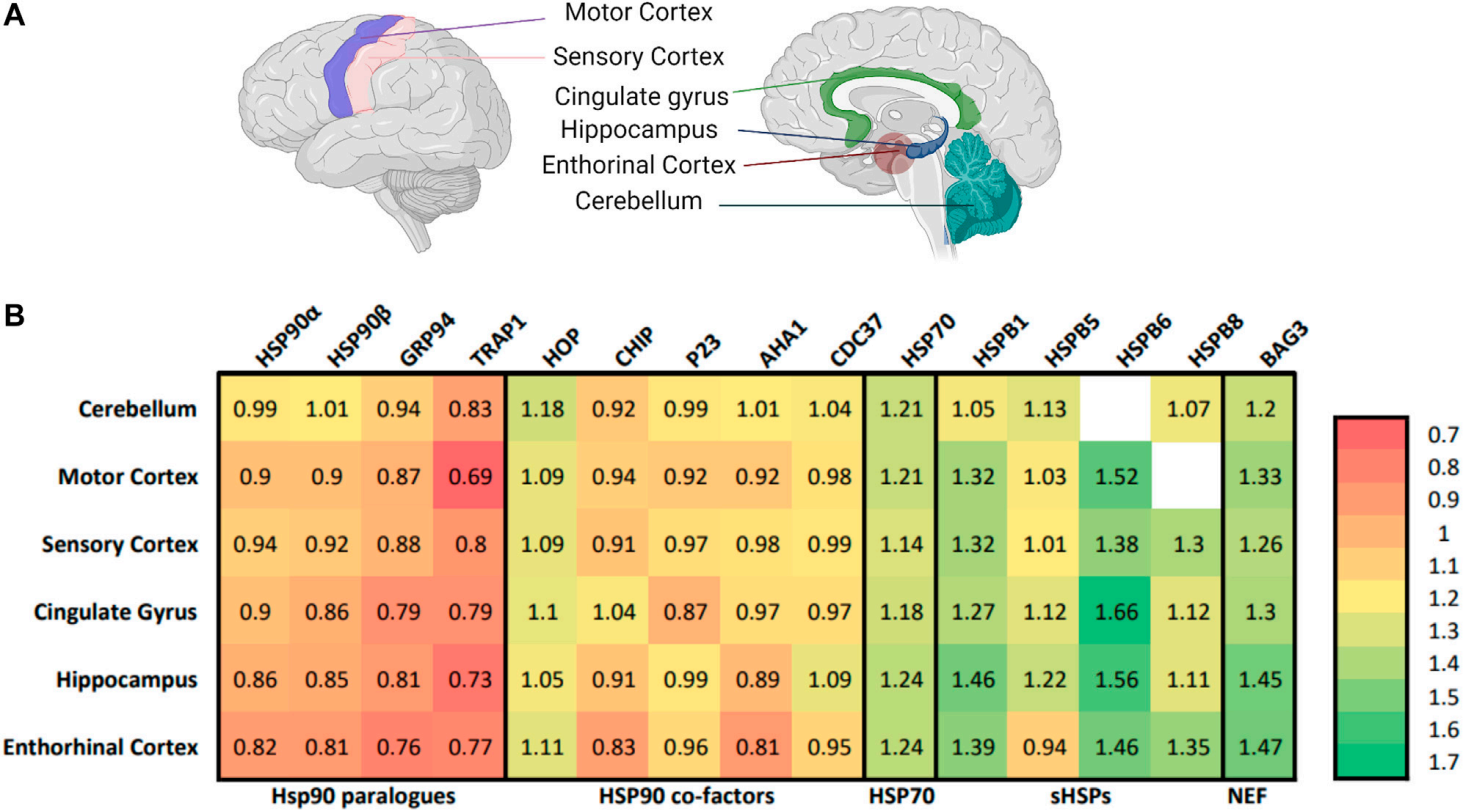
TRAP1 is down-regulated in different areas of the AD-brain. **(A)** The selected brain regions for proteomic analysis comparing patients with AD to non-AD counterparts (Xu et al., 2019). On the left, the lateral view of the left side of the brain, which contains the motor cortex and the sensory cortex. On the right the sagittal cut, revealing the cingulate gyrus, hippocampus, enthorinal cortex and cerebellum. The cingulate gyrus, hippocampus and enthorinal cortex are selected to represent heavily affected areas of the brain, the motor cortex and sensory cortex are less affected, while the cerebellum seems to be spared. Figure made with BioRender. **(B)** Heat map with the relative expression of elements of the protein quality control in the AD-brain compared to non-AD counterparts in different areas of the brain. Numbers represent the relative expression of that protein in an AD-patient compared to a non-AD counterpart (Koopman and Rüdiger, 2020). Included are the Hsp90 family, co-chaperones of HSP90, small heat shock proteins and nucleotide exchange factors. Notable is that the whole HSP90 family is affected in AD, with TRAP1 showing the largest decline in all areas of the brain.

The AD-brain shows distinctive alterations in the protein quality network, including downregulation of all four paralogues of the major chaperone family: Hsp90 (Xu et al., 2019; Koopman and Rüdiger, 2020). Molecular chaperones, such as Hsp90s, are proteins that assist in and control protein folding and unfolding in the cell. Notably, the brain tissues most severely affected in AD, the hippocampus, entorhinal cortex and cingulate gyrus, show the strongest alterations in Hsp90 levels (Figure 1A). Remarkably, although Tau aggregation of AD is taking place in the cytosol, it is the mitochondrial tumor necrosis factor receptor-associated protein 1 (TRAP1) that is most severely down-regulated in all regions of the brain, with a reduction of 31% in the sensory cortex, 27% in the hippocampus and 21% in the cingulate gyrus (Figure 1B) (Koopman and Rüdiger, 2020). For comparison, its cytosolic paralogues show the strongest reduction in levels of up to 19% in the entorhinal cortex (Koopman and Rüdiger, 2020).

The Hsp90 chaperone family is present in the main folding compartments of the cell, cytoplasm, ER and mitochondria, and its members are linked to AD in various ways (Koren III et al., 2009; Blair et al., 2014). Hsp90 mediates for example the transcription of the precursor of Aβ and proteins involved in synaptic plasticity (Chen et al., 2014). Most importantly, cytosolic Hsp90 controls Tau levels. *Via* cooperation with the E3 ubiquitin ligase CHIP, it is involved in targeting Tau for proteosomal degradation in healthy neurons (Dickey et al., 2007).

**FIGURE 2:**
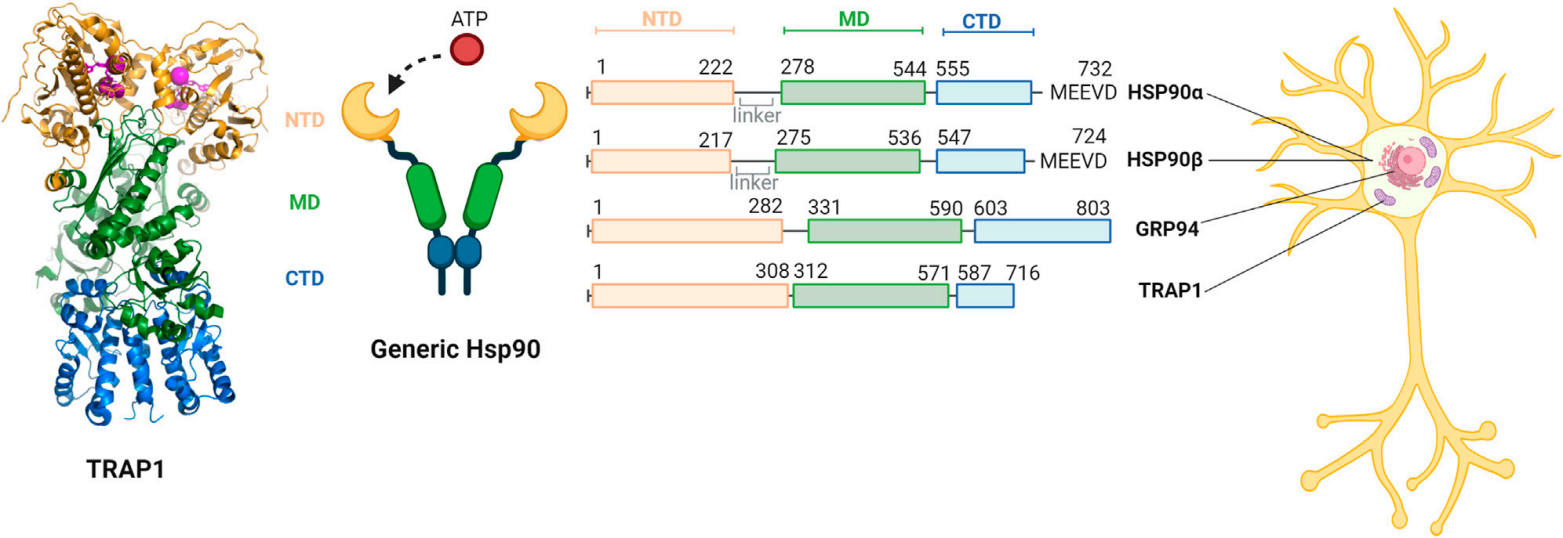
Comparison of the domains of members of the human Hsp90 family and their relative cellular compartments. The family members each have a N-terminal domain (NTD, yellow) responsible for ATP binding, a C-terminal domain (CTD, blue) responsible for dimerisation, and a middle domain (MD, green). A generic structure of the open confirmation of Hsp90 is provided. HSP90α and HSP90β are located in the cytosol and contain a linker and the MEEVD motif that recognises a large part of their clients. GRP94 is located in the endoplasmic reticulum and does not contain a linker nor the MEEVD motif. TRAP1 is primarily located in the mitochondria does not have a linker or the MEEVD motif either. The structure of the TRAP1 dimer (PDB code 6XG6) is depicted on the left, with the domains in the represented colors and cofactors and ADP in lila (Liu et al., 2020). Figure made with BioRender.

The Hsp90 family is highly conserved in organisms varying from bacteria to mammals (Felts et al., 2000). The acronym HSP refers to the historic discovery as Heat Shock Protein, and indeed some Hsp90s also act as stress proteins, which are upregulated by elevated temperatures, aggregates or other cell stress (Schopf et al., 2017). Hsp90s are also involved in cell survival, regulating apoptosis and transporting client proteins (Landriscina et al., 2010; Radli and Rüdiger, 2018; Schopf et al., 2017). The family consists of four paralogues which are predominantly available in their own cellular compartments (Figure 2) (Lettini et al., 2017). The two cytosolic paralogues, the stress-inducible HSP90α and constitutively expressed HSP90β, have a homology of 86% (Moore et al., 1989). GRP94 is located in the endoplasmic reticulum (ER) and is 50% identical to both HSP90s (Lettini et al., 2017). TRAP1 resides predominantly in mitochondria (Cechetto and Gupta, 2000). It is 60% similar to both HSP90s (Lettini et al., 2017). Interestingly, the mitochondrial function has been considered to be severely compromised in AD (Hirai et al., 2001; Cenini and Voos, 2019), which further inspires to have a closer look at a possible role of TRAP1 or its clients in AD.

With this review, we aim to investigate the effect of the distortion of TRAP1 levels and its possible role in AD. We describe the Hsp90 family and how it functions in the PQC system, and we discuss mechanism and structure of TRAP1 in particular. We examine its role in mitochondrial PQC and it simplication for metabolism, ROS reduction and mitochondrial pore opening. Then, we look at the effects TRAP1-decline may have on the cell, such as stalk regulation and mitochondrial-ER crosstalk, and we discuss mechanisms to regulate this chaperone. Finally, we put the cellular function of TRAP1 in context to its downregulation in AD brain tissue and discuss potential implications.

## TRAP1: Family and Function

Like all Hsp90 paralogues, TRAP1 consists of three conserved domains: the N-terminal domain (NTD), a middle domain (MD) the C-terminal domain (CTD) (Figure 2) (Kang, 2012). The CTD is responsible for inherent dimerisation, while the NTD can bind ATP and undergoes transient dimerisation (Prodromou, 2016). The first 59 amino acids of the N-terminus comprise the mitochondrial signal sequence. As many other matrix chaperones, the mechanism of action of TRAP1 is closely related to the bacterial homologue, HtpG (Shiau et al., 2006). Both TRAP1 and HtpG do not have any co-chaperones, unlike the cytosolic HSP90s (Zuehlke and Johnson, 2010). Additionally, both TRAP1 and HtpG lack a charged linker region that is present in all other forms of Hsp90 (Cechetto and Gupta, 2000). The bacterial homologue may thus present a good comparison for TRAP1.

In contrast to other Hsp90 paralogues, TRAP1 can form tetramers, as dimer of dimers (Joshi et al., 2020). Also, the bacterial HtpG can form tetramers, although it is unclear whether they have physiological relevance (Shiau et al., 2006). For TRAP1, however, there is a functional implication of tetramer formation, related to a role in metabolic regulation in the mitochondria (Joshi et al., 2020). Inhibition of the N-terminal ATPase of TRAP1 supports tetramer formation, indicating that modulation of the quaternary structure may be controlled by the functional cycle.

### Client Recognition by the Hsp90 Family

Hsp90 chaperones support protein folding downstream of Hsp70 chaperones (Morán Luengo et al., 2018; Genest et al., 2019). Hsp90 releases an Hsp70-inflicted folding block, which promotes folding to the native state (Morán Luengo et al., 2018; Morán Luengo et al., 2019). In the eukaryotic cytosol, a plethora of co-chaperones recruits Hsp90 to specific tasks, in particular supporting folding and maturation of signalling proteins such as kinases and steroid receptors (Schopf et al., 2017). Strikingly, such co-chaperones are absent in mitochondria, and so are steroid receptors. The general substrate binding properties of the Hsp90 family, however, are conserved (Liu et al., 2020). Hsp90 family members do not recognise specific short sequence motifs, but instead scattered hydrophobicity as it is found in late folding intermediates or certain disordered proteins such as Tau or α-synuclein (Karagöz et al., 2014). Thus, binding to Hsp90 depends on the folding state of a protein (Radli and Rüdiger, 2018).

A cryo-EM structure of TRAP1 in complex with a 160 residues long N-terminal fragment of succinate dehydrogenase B (SdhB) and ATP provides insights into the molecular nature of TRAP1 substrate recognition (Liu et al., 2020). The TRAP1 dimers forms a horseshoe, in which the CTDs connect both halves and the NTDs form the tips (Figure 2). In the complex with SdhB1-160, the TRAP1 horseshoe is twisted around its own axis and the ATP-bound NTDs touch each other. A 21 residues long C-terminal stretch of the SdhB fragment (K137-L157) passes through the central cavity of the twisted horseshoe, while its N-terminal portion forms a globular N-terminal domain with little contact to TRAP1.

The central cavity of TRAP1 needs to bind to a diverse range of substrate proteins. The TRAP1-SdhB1-160 complex may disclose some general properties of TRAP1 specificity. This stretch has a content of 42% large hydrophobic (3 Leu, 1 Ile, 1 Val) or aromatic amino acids (1 Phe, 3 Tyr). This ratio of 1 in 2.4 residues is higher than average hydrophobicity in protein sequences (1 in 3.6) (Karagöz et al., 2014). Notably, only Tyr147 is involved in a specific interaction with a hydrophobic residue in TRAP1.

In the closed state, the overall substrate interaction is similar to that of cytosolic Hsp90 with the kinase Cdk4 and or with the ligand binding domain of glucocorticoid receptor (Verba et al., 2016; Noddings et al., 2020). In TRAP1 the substrate stretch trapped inside the horseshoe is partially helical, in the other cases it is unfolded. This indicates that this substrate binding mode is conserved between paralogues, including some build-in adaptability to a broad range of substrates. The TRAP1 complex is symmetric, the Hsp90 complexes with Cdk4 and the ligand binding domain are not, which may reflect more the spectrum of conformational diversity of Hsp90 clients than possible paralogue specific differences in specificity.

### The TRAP1 ATPase Cycle

Hsp90 chaperones are ATPases, and the ATPase cycle regulates substrate influx, in particular substrate takeover from Hsp70 (Karagöz et al., 2014; Kirschke et al., 2014). ATP induces conformational changes that may influence the conformation of the bound client protein (Verba and Agard, 2017). The mechanism of action of TRAP1 follows a distinct cycle, which somewhat differs from the other Hsp90 paralogues (Matassa et al., 2012). TRAP1 adopts a closed-state conformation upon ATP binding (Leskovar et al., 2008). This is like the bacterial homologue, HtpG, while the eukaryotic cytoplasmatic paralogues require additional action of co-chaperones to induce the closed state (Leskovar et al., 2008). ATP closure leads to additional dimerization of the N-terminal domain *via* a coiled-coil interaction (Lavery et al., 2014). A two-step hydrolysis follows, and the dimer adopts an asymmetric conformation (Lavery et al., 2014). After ATP hydrolysis in one protomer, the dimer flips and remains in a closed state for the hydrolysis of the second ATP (Elnatan et al., 2017). This asymmetry is linked to client binding (Sima and Richter, 2018).

Compared to cytosolic HSP90, the affinity of TRAP1 for ATP is one order of magnitude higher (Leskovar et al., 2008). The ATP turnover rate activity, however, is comparable to other Hsp90s (Sima and Richter, 2018). ATPase activity can be inhibited by small molecule pocket antagonists, such as geldanamycin and radicicol (Altieri et al., 2012; Matassa et al., 2012). These antagonists can also bind HSP90, which makes specific inhibition difficult (Blair et al., 2014). Interestingly, the ATPase activity is inversely correlated with client recognition by TRAP1, which might be because of a recognition pattern nearby the ATP site (Joshi et al., 2020).

As for many other ATPases, bivalent cations support binding of the nucleotide by coordinating the phosphate groups (Elnatan et al., 2017). Interestingly, while most Hsp90s only employ magnesium, TRAP1 can use both magnesium and calcium (Elnatan and Agard, 2018). Calcium only is not enough to support hydrolysis in other Hsp90s (Elnatan and Agard, 2018). Contrary to magnesium, calcium is able to act cooperatively, while magnesium acts noncooperatively (Elnatan and Agard, 2018). The underlying mechanism on ATPase behaviour may thus be different compared to HSP90 (Elnatan and Agard, 2018).

### TRAP1 Lacks Co-Chaperones

Hsp90 chaperones act downstream of Hsp70 to break a folding block (Morán Luengo et al., 2018). This is an evolutionary conserved function of Hsp90s, established for the bacterial Hsp90, human HSP90α and the endoplasmic Grp94 (Pratt and Toft, 2003; Street et al., 2011; Morán Luengo et al., 2018; Genest et al., 2019; Morán Luengo et al., 2019). Given the conservation of the Hsp90 machine it is most likely this basic function also applies to TRAP1.

A major evolutionary difference between Hsp90s is regulation by co-chaperones. In the eukaryotic cytosol, Hsp90s are accompanied by co-chaperones that regulate the ATP cycle (e.g., p23 and Aha1), target Hsp90 towards specific substrates (e.g., Cdc37/p50 and Sgt1) or connect the machine *via* its C-terminal MEEVD motif to other cellular systems (e.g., CHIP and Hop; Blair et al., 2014). The mitochondrial TRAP1 lacks all of these, but so does the bacterial HtpG and the endoplasmic Grp94 (Masgras et al., 2017b). Thus, the bacterial HtpG represents a more suitable molecular paradigm to understand the mechanism of TRAP1 than the eukaryotic Hsp90. Like HtpG, the TRAP1 dimer closes immediately upon ATP binding (Elnatan et al., 2017; Schopf et al., 2017).

## TRAP1 and Mitochondrial Function

In contrast to its ubiquitously expressed paralogues, the expression of TRAP1 is largely restricted to the brain and testis, reflecting that both tissues are closely related (Guo et al., 2003; Kang et al., 2007). TRAP1 supports folding and assembly of factors involved in the energy metabolism. TRAP1 knock-out (KO) induces an increase in mitochondrial respiration and ATP production, resulting in continuous exposure to an elevated level of oxidative stress (Amoroso et al., 2014; Joshi et al., 2020). TRAP1 KO-mice show similar phenotypes with decreased mitochondrial function (Yoshida et al., 2013; Zhang et al., 2015). Consequently, decreased levels of TRAP1 show increased mitochondrial apoptosis (Altieri et al., 2012). Conversely, increased expression comes with reduced cell death, which can be linked to the up-regulation of TRAP1 in tumours of various kinds (Siegelin, 2013). Notably, also cancer cells express TRAP1, which enhances cell proliferation (Kang et al., 2007; Agorreta et al., 2014). In fact, the role of TRAP1 and other Hsp90 chaperones in cancer is extensively studied and better understood than TRAP1 action in neurodegeneration (Butler et al., 2015; Yuno et al., 2018; Taldone et al., 2020). TRAP1 acts as a cytoprotector of the mitochondria, based on three functions (Kang, 2012): 1) TRAP1 is crucial in inhibiting cell death by inhibiting CypD induced cytochrome C release into the cytosplasm (Altieri et al., 2012). 2) TRAP1 plays a major role in the mitochondrial PQC, loss of this control leads to autophagy (Siegelin et al., 2011; Labbadia et al., 2017). 3) TRAP1 disruption induces a metabolic shift towards glutamine metabolism, which points to TRAP1’s regulatory role in oxidative phosphorylation (OXPHOS) (Joshi et al., 2020).

### TRAP1 Rewires the Metabolic System

**FIGURE 3:**
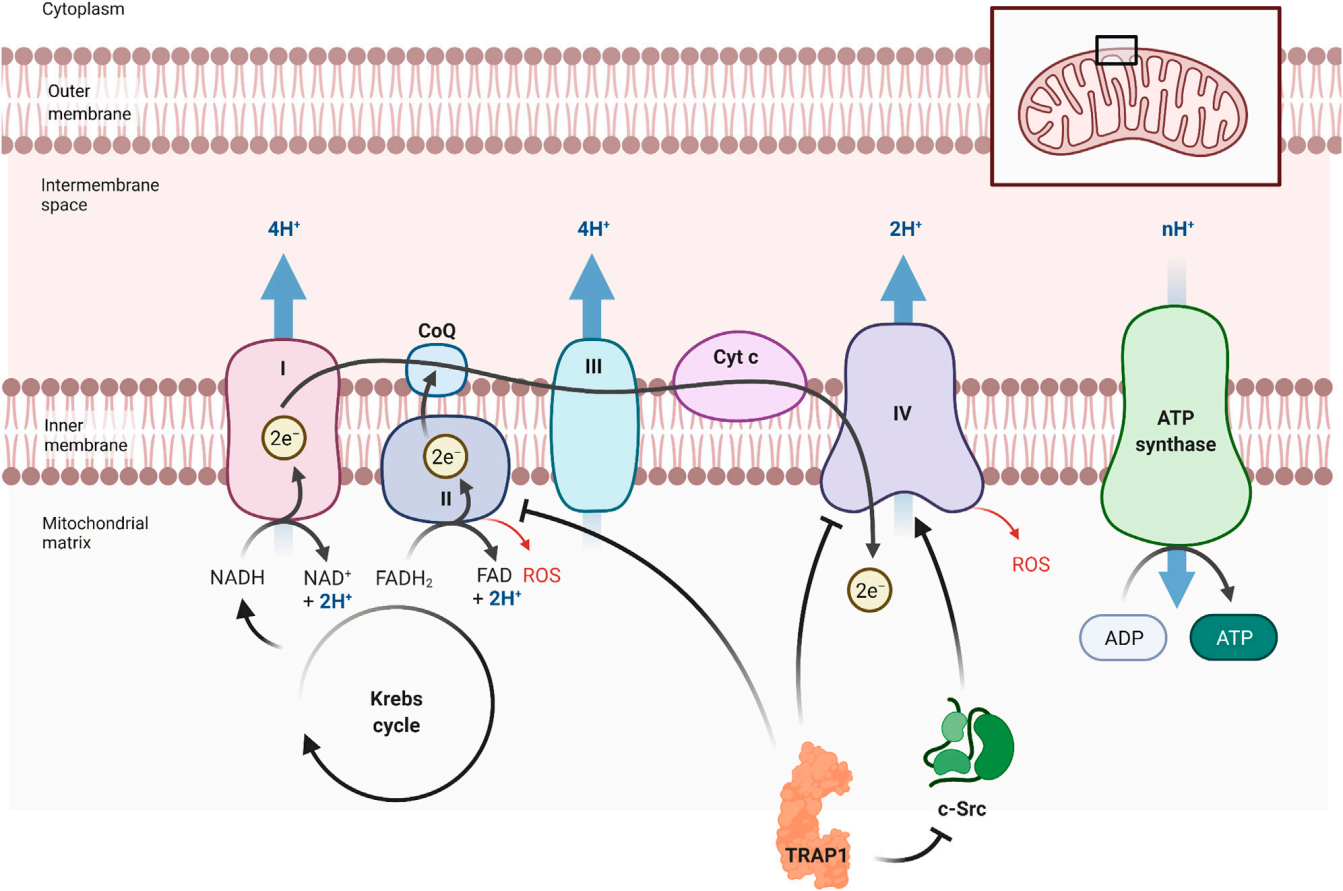
TRAP1’s influence on the electron transport chain. TRAP1 can influence metabolism, preferring glycolysis over OXPHOS (Joshi et al., 2020). It inhibits complex II and complex IV, which are essential in OXPHOS. TRAP1 can also inhibit Proto-oncogene tyrosine-protein kinase (c-Src), resulting in more inhibition of complex IV. By reducing OXPHOS, less ATP is produced but also less ROS are formed. This also promotes the production of ATP under oxygen shortage. Figure made with BioRender.

TRAP1 interacts with proteins of the mitochondrial electron transport chain (ETC), which is part of the ATP synthesis cascade (Figure 3) (Joshi et al., 2020). Being the only Hsp90 in the mitochondria makes TRAP1 destined as prominent chaperone on PQC of the energy metabolism. Indeed, TRAP1 acts on the metabolic balance between oxidative phosphorylation (OXPHOS) and aerobic glycolysis (Yoshida et al., 2013; Joshi et al., 2020). The chaperone may be seen as part of a metabolic switch that favours glycolysis over OXPHOS, which occurs often upon shortage of oxygen and is also known as the Warburg effect (Rasola et al., 2014). The upregulation of glycolysis has major consequences for the cell. First, mitochondrial respiration is decreased, as OXPHOS produces relatively more energy. Second, ROS, mainly produced during OXPHOS, are decreased. Third, more NADPH becomes available, which is an important ROS scavenger, thereby reducing ROS even further (Rasola et al., 2014). In fact, ROS contribute to the onset of AD and other neurodegenerative diseases (Morán et al., 2012; Ahmad et al., 2017; Cenini and Voos, 2019).

The mechanism behind OXPHOS regulation can be explained by the association of TRAP1 with components of the ETC (Figure 3). The most important TRAP1 effects on the ETC are through the interaction with complex II (SDH) and complex IV (Rasola et al., 2014; Joshi et al., 2020). Complex II is important in regulation of the energy metabolism of the ETC, depending on the quaternary structure of TRAP1 (Joshi et al., 2020). Recent findings show that TRAP1 activity stabilises or inhibits complex II, providing a unifying concept for apparently contradictory findings (Rasola et al., 2014; Guzzo et al., 2014). Subunit B of SDH stabilizes TRAP1 dimer closure, which in return stabilized the folding intermediates of SdhB (Liu et al., 2020). Interaction of TRAP1 with subunit A of SDH, however, reduces SDH activity, resulting in less electron funnelling and lower ROS generation (Guzzo et al., 2014).

AD-related oxidative damaged is attributed to decreased complex IV activity (Morán et al., 2012). TRAP1 inhibits complex IV activity through direct interaction with the complex and interaction with mitochondrial Proto-oncogene tyrosine-protein kinase (c-Src) (Yoshida et al., 2013). c-Src is well-known as a membrane-associated kinase but can also reside in the mitochondria (Miyazaki et al., 2003; Ogura et al., 2012; Yoshida et al., 2013). This kinase can phosphorylate both complex IV as well as TRAP1 (Yoshida et al., 2013). By both direct and indirect—through c-Src—interaction, TRAP1 shifts metabolic balance to aerobic glycolysis, which impairs metabolic respiration (Yoshida et al., 2013; Rasola et al., 2014). HSP90 interacts with cytosolic c-Src, resulting in the maturation of this kinase, while c-Src itself phosphorylates Tyr-416 in the activation loop of HSP90 (Xu et al., 1999). TRAP1 may interact with mitochondrial c-Src in a similar fashion.

It may seem counter-intuitive that an Hsp90 chaperone should inhibit ETC complexes. This, however, would be similar to the function of its cytosolic paralogues. As Hsp90s are tailored to bind to late folding intermediates, formation of a stable complex with an Hsp90 family members may be designed to maintain a protein required for a specific switch on an inactive form (Schopf et al., 2017; Radli and Rüdiger, 2018). It than may only require a minimal modification to unleash an active protein changing the programme of the cell. Cytosolic examples are the Hsp90 complexes with Cdk4 and GR-LBD (Verba et al., 2016; Radli and Rüdiger, 2018; Noddings et al., 2020).

### TRAP1 Protects Mitochondria From Oxidative Stress

A side reaction in the ETC leads to the production of reactive oxygen species (ROS) (Wang et al., 2013; Gaki and Papavassiliou, 2014). Since the mitochondria are the main source of ROS production, it is also the cellular compartment that suffers the most damage from these particles due to the reactivity of these particles with nearby tissue (Wang et al., 2013; Avolio et al., 2020). Once damaged, the efficiency of the ETC declines, amplifying ROS production (Wang et al., 2013; Avolio et al., 2020). In fact, elevated oxidative stress and mitochondrial dysfunction are early events in AD and potentially preceding protein aggregation (Ahmad et al., 2017).

**FIGURE 4:**
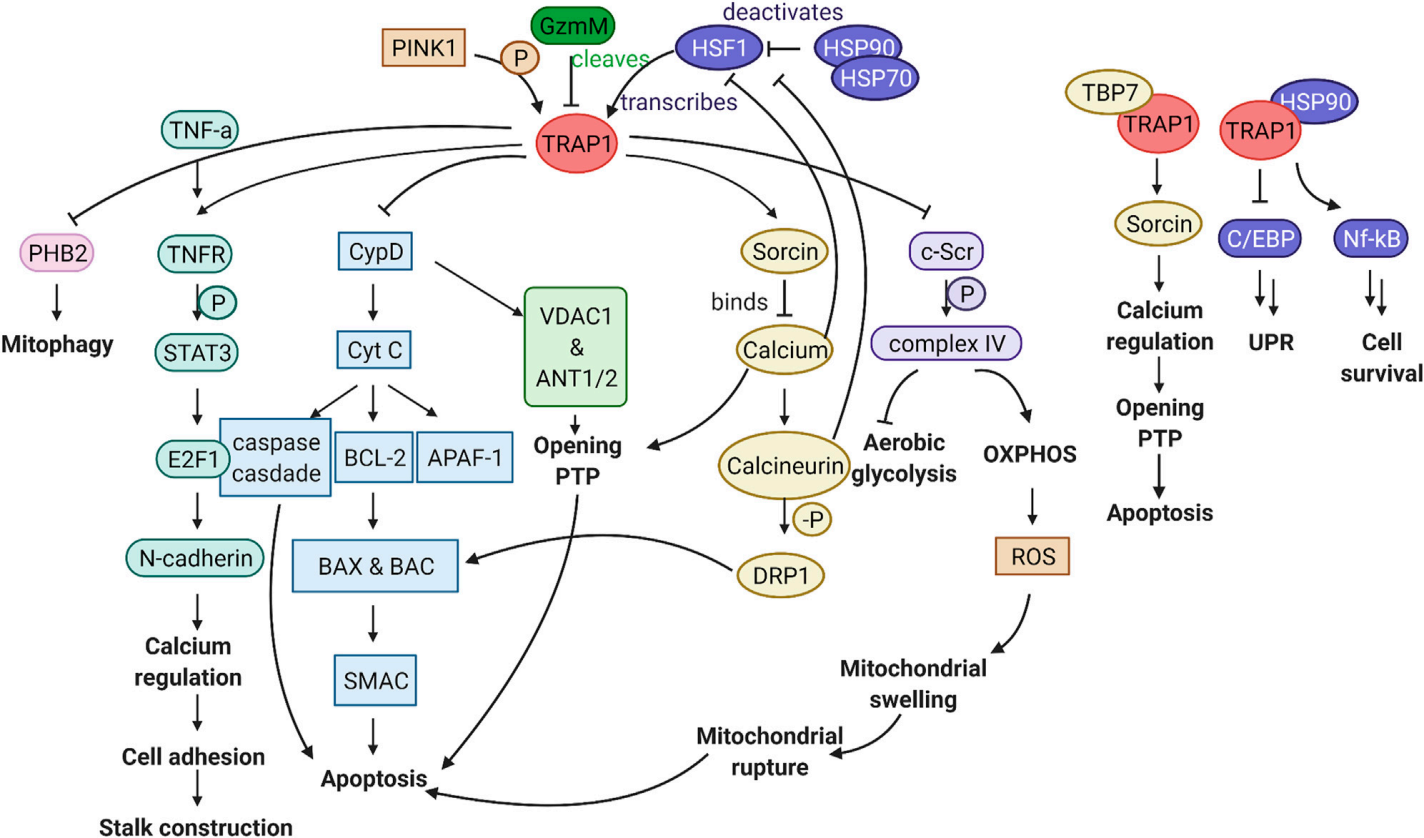
Graphical abstract of the TRAP1 pathway. TRAP1 is transcribed by HSF1, which is deactivated by the HSP90 complex. PINK1 phosphorylation can activate TRAP1, whereas GzmM can cleave TRAP1, thereby hindering TRAP1 activity. Downstream of TRAP1, multiple pathways can influence mitochondrial function and cellular viability. FLTR:TRAP1 interactor PHB2 is inhibited, reducing mitophagy. Stalk construction can be mediated through TNFR1, *via* STAT3 phosphorylation, activation of transcription factor E2F1, which transcribes N-cadherin responsible for calcium regulation and cell adhesion. Through inhibition of CypD, TRAP1 can prevent mitochondrial pore opening and release of Cyt c to the cytosol, which is critical in the prevention of apoptosis. TRAP1 can influence calcium regulation through Sorcin, which is essential to keep the mitochondrial pores close. By inhibiting c-Scr, TRAP1 acts as a metabolic switch, which results in less ROS production. In association with TBP7 on the ER, TRAP1 can influence calcium regulation between the mitochondria and ER. Together with HSP90, TRAP1 is involved in inhibition of the unfolded protein response and promoting cell survival. Figure made with BioRender.

TRAP1 levels are inversely correlated with the presence of ROS. Overexpression of TRAP1 leads to a decrease in ROS, whereas TRAP1 decrease, induced with iron chelator deferoxamine (DFO), leads to ROS accumulation (Im et al., 2007). A link between TRAP1 and Granzyme M (GzmM) further strengthens the relationship between TRAP1 and ROS (Figure 4) (Hua et al., 2007). GzmM is a serine protease which is important in the induction of cell death by cleavage of its client proteins (De Poot and Bovenschen, 2014). GzmM cleaves TRAP1, after which cytochrome C is released. This release induces caspase-mediated apoptosis. Ultimately, this leads to mitochondrial swelling, loss of the mitochondrial membrane potential, and subsequently activates the mitochondrial apoptotic machinery (Baines et al., 2005; Hua et al., 2007).

### TRAP1 is Key to Mitochondrial Homeostasis

**FIGURE 5:**
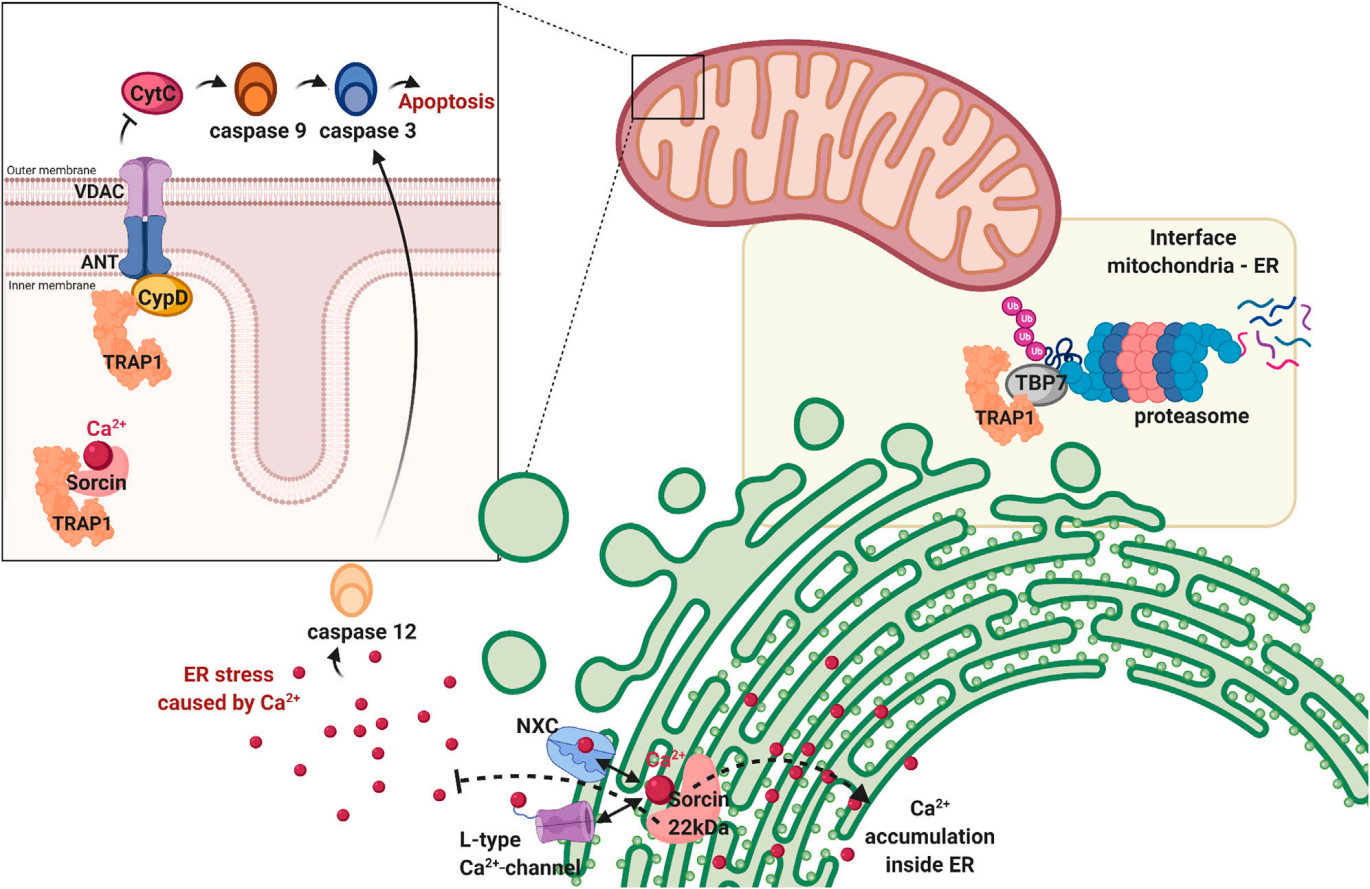
TRAP1 is involved in maintaining cellular homeostasis. First, TRAP1 regulates mitochondrial homeostasis by preventing the permeability transition pore formed by VDAC and ANT from opening by antagonising cyclophilin D (CypD). When this pore is opened, Cytochrome C (CytC) is released in the cytosol, activating the caspase cascade resulting in apoptosis. Second, TRAP1 is also found on the interface of the mitocohondria and endoplasmic reticulum, associating with tat-binding protein 7 (TBP7), a subunit of the proteasome. It helps regulating protein homeostasis before they enter the mitochondria, repairing if possible or degrading proteins if damaged beyond repair. Third, the dual location of the sorcin-isoforms suggests TRAP1 to have a role in calcium regulation (represented as red dots). Sorcin is a calcium sensor, which can regulate Na^+^/Ca^2+^ channel (NXC) and L-type Ca^2+^ channel on the endoplasmic reticulum, maintaining calcium homeostasis within the organelle and preventing cellular stress resulting from cytosolic calcium. Sorcin is a TRAP1 client and is thought to have a similar role in the mitochondria. Figure made with BioRender.

TRAP1 also has a general function in maintaining mitochondrial homeostasis (Altieri et al., 2012). TRAP1 is involved in the regulation of the permeability transition pore (PTP) (Figure 5) (Altieri et al., 2012). The mitochondrial PTP consists of the adenine nucleotide translocator (ANT1/2), the voltage-dependent anion channel (VDAC1) and the peptidyl-prolyl *cis*-trans isomerase (PPIase) cyclophilin D (CypD) (Baines et al., 2005). Opening of the inner mitochondrial membrane results in a loss of the mitochondrial membrane potential, followed by swelling, rupture of the membrane and apoptosis (Kang, 2012). The PTP is directly related to perturbations in cells and synapses seen in AD (Du et al., 2008). Silencing TRAP1 induces mitochondrial PTP opening while up-regulation has the opposite effect (Xiang et al., 2010).

The mechanism behind this inhibition of pore formation can be explained by inhibition of TRAP1 by CypD. CypD is a mitochondrial matrix protein responsible for the formation of pores in the mitochondrial inner membrane (Kang, 2012). Upon activation, it switches the conformation of ANT1/2 and VDAC1, thereby creating non-selective pores (Kang et al., 2007). Hsp90s can bind CypD before it is fully folded, which is likely cause of the inhibitory effect. Upon release from Hsp90, CypD will release Cyt C into the cytosol, inducing cell death (Siegelin, 2013). TRAP1 prevents pore-opening by blocking this conformational switch of CypD. Similar action can be performed by HSP90, which enters the mitochondria only under special circumstances, such as in cancer (Kang, 2012). Protein interaction between TRAP1/HSP90 and CypD maintains after the addition of Hsp90 inhibitors, which suggests a different interaction than that of the inhibitors (Kang, 2012). CypD-deficient mitochondria are resistant to mitochondrial swelling caused by Aβ and Ca^2+^ (Du et al., 2008). Deficiency also leads to improved synaptic function in AD-mice models (Du et al., 2008). TRAP1 inhibition of CypD may thus prove to be important in reducing some of the characteristics seen in AD.

Hence, TRAP1 silencing leads to an increase in Cyt C in the cytosol, paired with elevated caspase-3 activity, which releases a cascade leading to apoptosis (Figure 4) (Xiang et al., 2019). TRAP1 over-expression decreases cleavage of caspase 3, and thus also factors downstream in this cascade, including caspase 9 and poly (ADP-ribose) polymerase-1 (PARP) (Agorreta et al., 2014). Interaction of TRAP1 with cyt C is regulated by phosphorylation. PTEN induced putative kinase 1 (PINK1) localises with TRAP1 in the mitochondria (Pridgeon et al., 2007). It phosphorylates TRAP1, which enables TRAP1 to inhibit Cyt C release through CypD (Pridgeon et al., 2007). Upon PINK1 depletion with siRNA, more cytochrome C is released, which correlates with the reduction in TRAP1 phosphorylation (Pridgeon et al., 2007).

## TRAP1 and Cellular Function

TRAP1 is primarily located in the mitochondria. Interestingly, a small fraction resides in the cytosol and on the ER membrane (Kubota et al., 2009; Matassa et al., 2012; Amoroso et al., 2014). This is remarkable as only few proteins function in multiple cellular compartments, and TRAP1 has a typical N-terminal targeting signal for mitochondria (Cechetto and Gupta, 2000). However, these extramitochondrial locations were found by multiple research groups (Cechetto and Gupta, 2000; Kang et al., 2007). Interactome data suggest that a considerable number of the client proteins of TRAP1 do not reside in the mitochondria (Kang, 2012). This raises questions on possible export mechanisms, which have not been elucidated yet. Alternatively, TRAP1 may not be fully targeted towards mitochondria. Dual targeting of mitochondrial proteins has a biological function for other proteins, too (Kalderon and Pines, 2014; Dik et al., 2016; Ben-Menachem and Pines, 2017).

### TRAP1 and the ER

The junction between the ER and the mitochondria is important for cell death regulation (Grimm, 2012). The ER-mitochondria interface is known to exchange molecules for apoptosis induction, among which cytochrome C (Grimm, 2012). Ca^2+^ is the most prominent signal between these organelles, it mediates the induction of apoptosis at high concentrations (Grimm, 2012). Calcium dysregulation is also a key component in the pathogenesis of AD, involved in hyperphosphorylationof Tau and increased Aβ formation (LaFerla, 2002). Now, TRAP1 is implied to be involved in calcium communication (Matassa et al., 2012; Amoroso et al., 2014; Park et al., 2020). Inhibition of TRAP1 increases Ca^2+^ discharge in the mitochondria, comparable to GRP94 in the ER (Park et al., 2020). When these concentrations get too high in the organelle, the PTP will open, releasing calcium to the cytoplasm (Siegelin, 2013).

TRAP1 directly interacts with Tat-binding protein 7 (TBP7) on the cytoplasmatic side of the ER membrane (Figure 5) (Amoroso et al., 2012). TBP7 is the regulatory subunit of the proteasome and monitors proteins with mitochondrial destination (Matassa et al., 2012), including the 18 kDa Sorcin B isoform and F1 ATPase β-subunit (Amoroso et al., 2014). TBP7 contributes to protein quality control by sensing the folding state of these proteins (Matassa et al., 2012). Only if folded, they are imported to the mitochondria. If not, they are degraded by the proteasome (Finley, 2009; Matassa et al., 2012).

An alternative role for extramitochondrial TRAP1 is attributed to protection from ER stress (Matassa et al., 2012). TRAP1 plays a key role in the inhibition of apoptosis, caused by the mitochondrial apoptotic machinery in response to strong ER stress (Park et al., 2020). Client protein Sorcin is hypothesized to be involved, as it resides in both mitochondria and ER. The 22 kDa Sorcin isoform resides in the ER and does not interact with TRAP1, the 18 kDa variant is in the mitochondria and is a client of TRAP1 (Matassa et al., 2012). Sorcin is a calcium sensor and regulates Ca^2+^ homeostasis through sodium-calcium exchanger (NXC) and the voltage dependent L-type Ca^2+^ channel (Figure 5) (Matassa et al., 2012). Accumulation of calcium in the ER is regulated by the 22 kDa isoform and prevents ER stress (Matassa et al., 2012). Considering the function of Sorcin ER paralogue, the 18 kDa isoform may have a role in Ca^2+^-homeostasis in the mitochondria, possibly by regulating the mitochondrial PTP.

### TRAP1 and the Cytoplasm

TRAP1 also has disease-relevant cytoplasmatic interactors (Cechetto and Gupta, 2000). The interaction between TRAP1 and the Retinoblasma protein (RB) was discovered in 1996 (Chen et al., 1996). RB is distributed over the cytoplasm, and TRAP1 forms a complex with it in the M phase and after heat shock (Chen et al., 1996). TRAP1 binds to the intracellular domain of TNFR, which gave TRAP1 its name (Song et al., 1995). TNFR is a transmembrane protein and can transmit the signals of extracellular TNF-α to the intracellular compartment, TRAP1 binds it intracellular domain (McCoy and Tansey, 2008; Cheng et al., 2014). After TRAP1 and TNFR1 interaction, TNFR1 phosphorylates signal transducer and activator of transcription 3 (STAT3), in the TNF-α/TNFR1 pathway (Figure 4) (Romanatto et al., 2007). STAT3 initiates E2F1 transcription, down-regulating N-cadherin in TRAP1 KO cells (Kubota et al., 2009). N-cadherin mediates cell-adhesion through calcium regulation, which leads to altered morphology of the neurons of TRAP1 KO cells, decreasing cellular communication (Kubota et al., 2009). This phenotypical change is also one perceived in AD-brains (Goedert and Spillantini, 2006; Perl, 2010). Hence, the perceived TRAP1 decrease in AD may further deteriorate cognitive function. Finally, also tumour suppressor EXT, an ER-resident transmembrane glycosyltransferase interacts with TRAP1 (Simmons et al., 1999). Remarkably, all these interactions take place in the cytoplasm. Again, this implies either an export mechanism for TRAP1, or an incomplete import after synthesising TRAP1 in the nucleus.

TRAP1 may also be directly involved in regulation of cytoplasmatic proteins by intercompartmental communication through activated pathways (Figure 4). TRAP1 has a central role in the intrinsic apoptotic pathway, releasing apoptogenetic proteins such as Cyt C to the cytosol (Altieri et al., 2012). This leads to the caspase cascade eventually inducing cell death. Calcium homeostasis is another way with which TRAP1 sends signals to the cytosol (Park et al., 2020). This mechanism is especially important for regulating the HSF1 transcription factor, which will be discussed below.

The third way of mitochondria-cytoplasm interaction of TRAP1 is through import of cytosolic proteins. Cytosolic HSP90α/β can be transported to the mitochondria, which is mainly seen in neurons and cancer cells (Kang et al., 2007; Workman and de Billy, 2007). How these predominantly cytosolic chaperones are imported, remains to be elucidated (Kang et al., 2007). In the mitochondria, HSP90 becomes part of the mitochondrial chaperone network and performs similar actions as TRAP1 on CypD function (Workman and de Billy, 2007). More importantly, HSP90 brings mitochondrial targeted preproteins to import receptor translocase of outer membrane 70 (Tom70) (Lin et al., 2015). These preproteins may well be interactors of another Hsp90 family member: TRAP1.

### The Effect of TRAP1 on the Proteome

Can altered TRAP1 expression affect the expression of other proteins? Silencing of TRAP1 causes only minor proteomic alteration, even of the proteins that interact directly with TRAP1, indicating that TRAP1 does not affect protein synthesis (Joshi et al., 2020). Notably, loss of cytosolic HSP90 does affect the proteome immensely, where TRAP1 does not (Joshi et al., 2020). This is comparable to HtpG, the bacterial Hsp90, which is non-essential in *E. coli* (Millson et al., 2004).

An exception is prohibitin (PHB2), a mitochondrial membrane protein that is more expressed upon TRAP1 inhibition (Joshi et al., 2020). PHB2 functions as a receptor that induces mitophagy: a process marking the mitochondria for degradation (Lahiri and Klionsky, 2017). Up-regulation of this protein may thus indicate as a mechanism to cope with TRAP1 loss.

Interestingly, TRAP1 inhibition leads to a significant increase in glutathione (GSH) (Joshi et al., 2020). This might be to compensate for the increase in ROS upon TRAP1 silencing. Unfortunately, GSH levels are also decreased in the AD-brain and cannot fulfill its role as antioxidant upon ROS increased (Saharan and Mandal, 2014). TRAP1 primarily interacts with 81 proteins, among which other mitochondrial proteins, OXPHOS complex subunits, channel and carrier proteins and mitochondrial enzymes (Joshi et al., 2020). Notable is that these interactions are inversely correlated with the ATPase activity of TRAP1 (Joshi et al., 2020). The association with the OXPHOS complex subunits supports the role of TRAP1 in mitochondrial respiration. However, proteins associated with glucose metabolism are not affected upon TRAP1 silencing (Joshi et al., 2020).

Key interactors include the mitochondrial proteins such as GRP75, CH60 and PHB2 (Joshi et al., 2020). Comparable proteins interact with the yeast variant of HSP90, which enlarges its credibility as a model for human TRAP1 (Millson et al., 2004). Some known partner proteins evade the proteomics analysis, many of which are low abundance regulatory proteins. These include the mitochondrial cyclophylin D, PINK1 and c-Src and the cytosolic type I tumour necrosis factor receptor (TNFR1), RB and EXT proteins.

## Regulation of TRAP1

Not only TRAP1, but also HSP90α/β and GRP94 have lower levels in patients with AD (Xu et al., 2019; Koopman and Rüdiger, 2020). These chaperones are all regulated by the same stress-inducible transcription factor, HSF1. In contrast, all HSP90 paralogues are severely upregulated in cancer (Park et al., 2020). Down-regulation of all Hsp90s in AD could contribute to the perceived cell death, while simultaneous inactivation of all paralogues is cytotoxic to cancer cells (Kang, 2012; Park et al., 2020).

### Transcription of TRAP1 by HSF1

**FIGURE 6:**
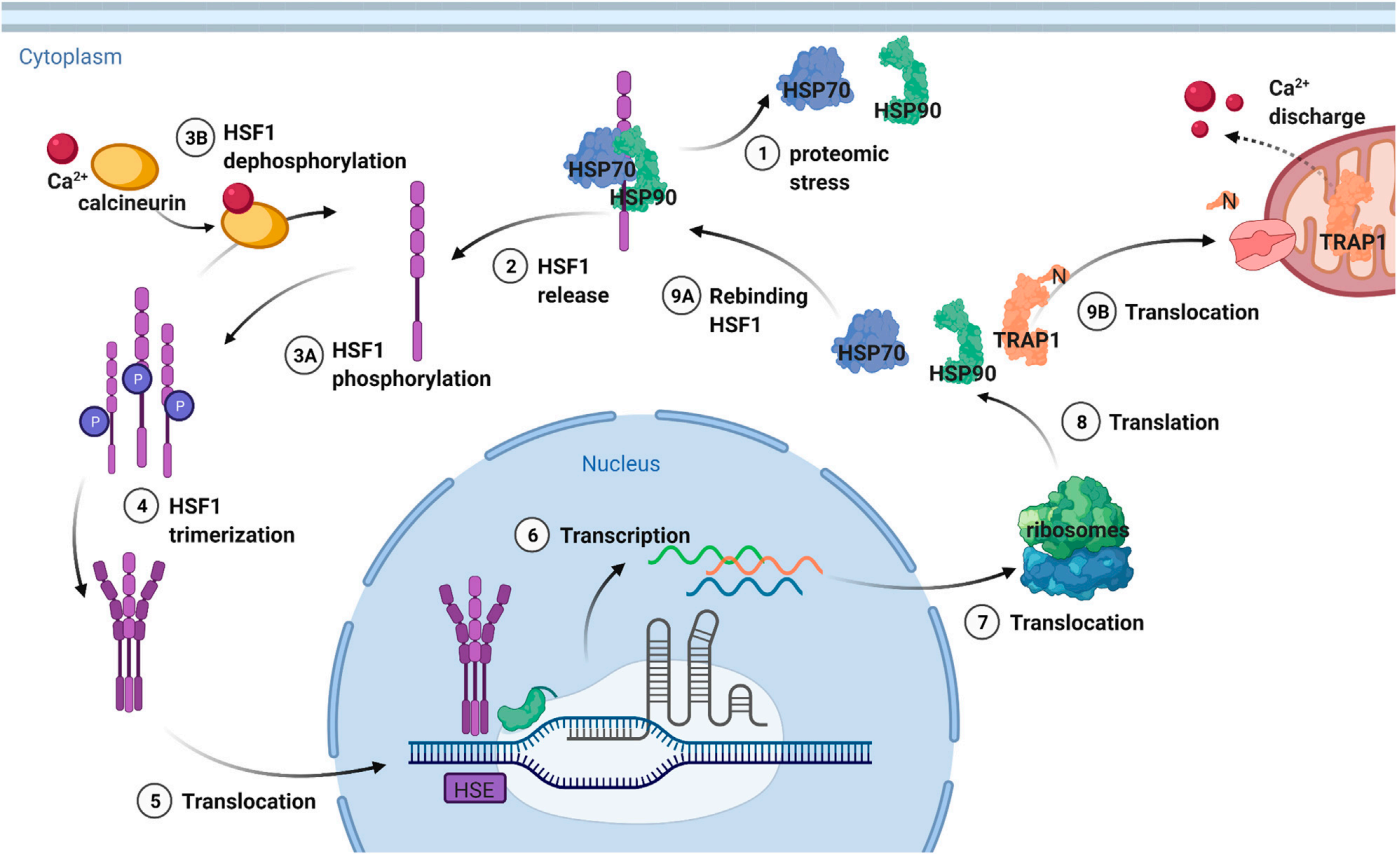
Transcription regulation of TRAP1 and other heat shock proteins by HSF1. HSF1 is deactivated by a complex formed from HSP70 and HSP90, which release the transcription factor upon proteomic stress. Then HSF1 is phosphorylated and can trimerize, after which it is translocated to the nucleus. The trimerization is needed for recognition of the heat shock elements (HSE) after which transcription takes place. The DNA is then translocated to the ribosomes that translate it to heat shock proteins. TRAP1 is then translocated to the mitochondria, where the N-terminal sequence targeting for this location is cleaved off. HSP90 and HSP70 stay in the cytosol, rebinding HSF1 and thereby providing a negative feedback mechanism. Figure made with BioRender.

TRAP1 transcription is regulated by heat shock factor-1 (HSF1) (Figure 6). HSF1 is involved in the regulation of the heat shock response, including the heat shock proteins HSP27, HSP40 and HSP70 and HSP90 (Workman and de Billy, 2007; Mahat et al., 2016; Schopf et al., 2017). It exerts most of its action by increasing RNA polymerase II release from promotor-proximal pause (Mahat et al., 2016). HSF1 can be rapidly conformationally activated upon proteotoxic stress such as heat shock, after which it regulates chaperone expression (Anckar and Sistonen, 2011; Mahat et al., 2016; Solís et al., 2016). This chaperone regulation by HSF1 according to fluctuating cellular requirements is preserved among eukaryotes (Pincus, 2020; Kainth et al., 2021). The levels of all Hsp90 chaperones are reduced in AD while HSP70 is overproduced (Koopman and Rüdiger, 2020). Thus, it is interesting that HSF1 expression is also lower in AD-phenotypical rats, and it will be interesting to understand the precise mechanism of the feedback regulation of heat shock protein expression (Jiang et al., 2013).

HSF1 is inactivated by a complex formed by HSP90 and HSP70 (Figure 6) (Workman and de Billy, 2007; Schopf et al., 2017; Park et al., 2020). The closed conformation of HSP90, which is the ATP bound conformation, interacts with HSF1 through its N-domain (Kijima et al., 2018). HSP70 binding functions as chaperone switch for HSF1 activity (Zheng et al., 2016) Upon proteotoxic stress, the intracellular concentration of misfolded proteins is increased (Labbadia et al., 2017). HSP90 and HSP70 are then needed to protect the cell from damage by unfolded client proteins. As a result, they dissociate from HSF1, which can now trimerize. Subsequently, HSF1 is hyperphosphorylated, which is a hallmark of the heat shock response, although not required for HSF1 activity (Zheng et al., 2016). Instead, differences in phosphorylation generate cell-to-cell differences in Hsp90 levels (Zheng et al., 2018) It is trimerized HSF1 that can bind to the heat shock elements (HSE) in the promotors of the target genes (Pincus, 2020).

HSF1 is then translocated from the cytoplasm to the nucleus, where it accumulates (Figure 6) (Anckar and Sistonen, 2011). PTMs play an important role in this translocation (Park et al., 2020). The dephosphorylation of HSF1 by calcineurin can negatively affect the migration of HSF1 (Park et al., 2020). Inhibition of this protein leads to an increase in migration, increasing expression of HSPs. In turn, calcineurin is activated by calcium, making this ion important for regulating HSF1 activity (Park et al., 2020). Calcium homeostasis is influenced by TRAP1, as calcium discharge by TRAP1 from the mitochondria suppresses the HSF1 regulated transcription of heat shock proteins (Park et al., 2020). Hence, inhibition of TRAP1 causes an increase in calcium release, activating calcineurin and decreasing HSF1 migration. In turn, TRAP1-KO increases transcription of mitochondrial chaperone genes by HSF1, which suggests HSF1 to be a potential guardian of mitochondrial function upon impaired proteostasis (Katiyar et al., 2020).

### Induction and Suppression of TRAP1 Expression

So, we can induce TRAP1 expression by promoting HSF1 transcription while TRAP1 provides negative feedback by promoting Ca^2+^ release from mitochondria. Agents inducing cell stress induce the HSR by inducing HSP90 release and HSF1 activation (Powers and Workman, 2007). For instance, proteasome inhibitors increase the concentration of misfolded proteins, which induces HSP90 release (Powers and Workman, 2007). Also, inflammatory agents such as Phospholipase A2 and arachidonate, promote increased DNA-binding by HSF1 (Powers and Workman, 2007). Stress can also be induced by disturbing the redox state of the cell, creating ROS. Finally, HSP90 inhibitors can also enhance HSF1 activity, by formation of repressive HSP90-complex.

The HSR can also be suppressed, e.g., by the non-specific agent quercetin (Powers and Workman, 2007; Workman and de Billy, 2007). Some agents can specifically reduce TRAP1 expression. β-Hydroxy-iso-valerylshikonin (β-HIVS) can induce a time-dependent decline of the amount of TRAP1 in the mitochondria (Masuda et al., 2004). Similarly, exposure to VP16, a DNA-damaging chemotherapeutic, reduces TRAP1 levels (Masuda et al., 2004). This reduction leads to apoptosis in most cells, through Cyt C release, as described above. The suppressing effects on TRAP1 of both compounds are counteracted by antioxidant N-acetyl-cysteine (NAC) (Masuda et al., 2004). In the presence of NAC, apoptosis is significantly decreased (Masuda et al., 2004). These findings further support to the role of TRAP1 in ROS reduction.

The primary phenotypic loss upon TRAP1 silencing is an increase in mitochondrial respiration and ATP production. Thus, TRAP1 functions as a negative regulator of mitochondrial OXPHOS (Joshi et al., 2020). OXPHOS is inhibited under hypoxia circumstances, which leads to an increase in TRAP1 expression (Joshi et al., 2020). ROS play a role in inducing and suppressing TRAP1. So, higher ROS levels suppress TRAP1 activity. ROS cause oxidative stress, which induces the transcription of heat shock proteins such as TRAP1 (Powers and Workman, 2007). The molecular mechanism of redox balance modulating TRAP1 levels remains to be elucidated.

### Modulation of TRAP1 Activity

Like all other Hsp90 paralogues, TRAP1 dimerises to form the catalytic active site needed for ATP hydrolysis (Elnatan et al., 2017; Elnatan and Agard, 2018). TRAP1, however, also forms tetramers as dimers of dimers in response to OXPHOS changes, which have been verified by three independent techniques (Joshi et al., 2020). Tetrameric TRAP1 may possibly be better suited to interact with larger mitochondrial complexes (Joshi et al., 2020). Exposure to OXPHOS increases expression of the tetramer, which suggests its functionality in regulating mitochondrial homeostasis (Joshi et al., 2020). Elevated temperatures also shift the equilibrium towards tetrameric TRAP1, possibly stabilising its structure (Joshi et al., 2020). The functional importance of this structure remains to be established, so are the dimer/tetramer balance and regulation mechanisms for this complex. It will be intriguing to identify the molecular function of this change in quaternary structure, and to which extent this may wider role beyond OXPHOS regulation.

PTMs play an important role in the regulation of TRAP1, including (de)acetylation, (de)phosphorylation, S-nitrosylation and ubiquitination (Table 1) (Anckar and Sistonen, 2011; Matassa et al., 2012; Faienza et al., 2020). Similar to HSP90, phosphorylation contributes to activation of TRAP1 (Trepel et al., 2010; Anckar and Sistonen, 2011). Phosphorylation is carried out by PINK1, but RK1/2 are also able to phosphorylate TRAP1, modulating TRAP1 metabolism (Masgras et al., 2017a). This phosphorylation enables a metabolic switch, promoting TRAP1 activity and cell survival (Masgras et al., 2017a). This switch in energy regulation equips cells to survive shortages of oxygen or nutrients, it is therefore common in cancer (Masgras et al., 2017a).

**TABLE 1:** Posttranslational Modifications (PTMs) of TRAP1. The different known PTMs for TRAP1. PTMs marked with a star* still need to be validated.

PTM	Amino acid	Enzyme	References
Phosphorylation	Y366, S401, T494, S511, S568	PINK1, ERK1/2	Koh and Chung. (2012); Matassa et al. (2012), Masgras et al. (2017a)
Acetylation	K87, K332, K382, K424, K466		Matassa et al. (2012)
Deacetylation	A	SIRT3	Faienza et al. (2020)
S-nitrosylation	C501, C573*	S-nitrosylase complexes	Faienza et al. (2020)

S-nitrosylation of Cys501 by S-nitrosylase complexes is associated with proteasomal degradation (Rizza et al., 2016). The cysteine can change the conformation of TRAP1 during its catalytic cycle, as determined by molecular dynamics simulations (Faienza et al., 2020). It also enables the interaction with client proteins (Faienza et al., 2020). This PTM has an inhibitory effect on the ATPase activity, which is in line with the protective role of TRAP1 in apoptosis (Faienza et al., 2020). Cys573 might be regulated by N-nitrosylation in a similar way, but this still needs to be validated (Faienza et al., 2020). S-nitrosylation inhibits chaperone activity of cytosolic Hsp90, similar to acetylation (Trepel et al., 2010; Matassa et al., 2012). It needs to be established whether these PTMs have comparable functions for TRAP1.

## TRAP1 Down-Regulation in AD

**FIGURE 7:**
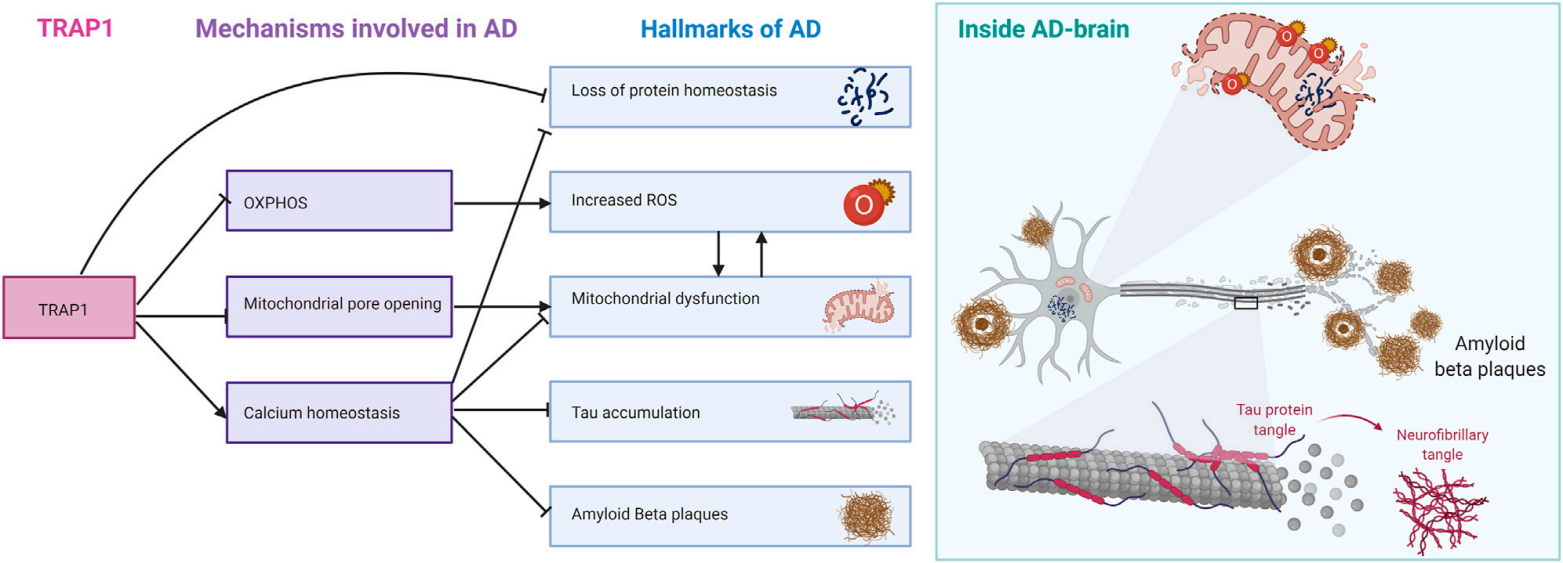
TRAP1 regulatory mechanisms linked to different hallmarks of AD. TRAP1 regulation of OXPHOS is linked to increased ROS formation, which damages mitochondria. Inhibition of mitochondrial pore opening by TRAP1 protects the mitochondria from swelling and rupture. TRAP1 is also involved in calcium homeostasis. Ca^2+^ proves to be important in signaling, and disrupting calcium homeostasis contributes to hallmarks such as Tau accumulation, formation of Amyloid Bèta Plaques and protein homeostasis. As mitochondrial chaperone, TRAP1 is also directly involved in maintaining protein homeostasis. On the right, the hallmarks of AD are summarised around one neuron.

TRAP1 is down-regulated in AD, together with all other Hsp90s (Koopman and Rüdiger, 2020). As Hsp90 is the only major chaperone family for which all members have reduced levels in disease in AD, it raises the how and why. Even more, as TRAP1 experiences the strongest reduction. While cytosolic Hsp90s interact with Tau, which aggregates in AD (Dickey et al., 2007). Such a connection would yet to be established for TRAP1. While we do not know whether TRAP1 is a driver or a passenger in AD progression, we can link some molecular functions of TRAP1 to pathogenetic hallmarks in AD (Figure 7). Even if TRAP1 may not be driving the disease itself, studying mechanism and function is likely disclose pathways that may have a key role in AD. The primary role found for TRAP1 is in mitochondrial dysfunction (Koh and Chung, 2012; Wang et al., 2013; Gaki and Papavassiliou, 2014; Joshi et al., 2020). TRAP1 seems to provide a dual role in protecting the mitochondria. First, the chaperone seems to have an essential role in the regulation of the metabolism of the ETC, switching from OXPHOS to glycolysis, thereby reducing ROS (Joshi et al., 2020). Second, TRAP1 can regulate mitochondrial PTP opening, and is therefore of importance in homeostasis (Siegelin, 2013). Decreased levels of TRAP1 may therefore contribute to contribute to the initial perceived oxidative stress and mitochondrial dysfunction in AD.

The role of ROS in Alzheimer’s Disease itself is ambiguous (Huang et al., 2016). While the general consensus is that oxidative stress is part of the pathology of AD, the exact role in the disease’s onset is unclear (Masuda et al., 2004; Siegelin, 2013; Ahmad et al., 2017; Umeno et al., 2017). The ability of TRAP1 to antagonise ROS production through its influence on the ETC is extraordinary (Hua et al., 2007; Im et al., 2007; Xu et al., 2009; Joshi et al., 2020). As a chaperone, it is not expected for TRAP1 to function as a regulator. Like bacterial HtpG, TRAP1 is not essential, suggesting effective backup systems for its function. Lastly, the observed effects upon silencing seem to be too small for TRAP1 to be a metabolic switch on its own. However, the observation of TRAP1 as a metabolic regulator is in line with another early pathogenic event: glucose hypometabolism (Du et al., 2018). It may be interesting to consider the link between the observed oxidative stress, mitochondrial dysfunction, and glucose hypometabolism through TRAP1. The role of TRAP1 in mitochondrial dysfunction strengthens the hypothesis that this protein may be of importance in AD.

It is not only on mitochondrial level that TRAP1 may contribute to the onset of AD. TRAP1 is significantly more expressed in the brain than in other tissues, suggesting a more important role in this body part (Kang et al., 2007). Silencing TRAP1 expression leads to synapses with reduced stalks, which affects neuronal communication (Kubota et al., 2009). Surprisingly, TRAP1 is found also outside of mitochondria. TRAP1 interacts on the ER-mitochondria interface with TBP7, thereby facilitating degradation of misfolded TRAP1 client proteins (Takemoto et al., 2011; Amoroso et al., 2012). Other client proteins of TRAP1 are cytosolic, such as RB, TNFR and EXT (Song et al., 1995; Chen et al., 1996; Simmons et al., 1999). Cytosolic localisation of TRAP1, however, is still matter of debate (Cechetto and Gupta, 2000; Joshi et al., 2020).

### TRAP1 and PQC Derailment in AD

So, lower levels of TRAP1 seem to have a significant effect on the functioning of neurons. But what is the cause of this decrease? Down-regulation of transcription factor HSF1 could explain the perceived lower levels of TRAP1 and other Hsp90s as well, which is already demonstrated in rats with AD-like phenotypes (Jiang et al., 2013). This of course would raise the question how this transcription factor could be downregulated in cells under so much stress as those in AD.

TRAP1 has been extensively investigated in the context of cancer (Workman and de Billy, 2007; Trepel et al., 2010; Siegelin, 2013; Masgras et al., 2017b). Here, TRAP1 and other chaperones are over-expressed, which contributes to the inhibition of apoptosis (Lettini et al., 2017). Induction of expression of TRAP1 in AD might exploit a similar principle to keep the neurons intact. However, many processes are derailed in cancer, not only the expression of chaperones. Hence, it is difficult to pinpoint which derailment leads exactly to which cellular event. The role TRAP1 in AD is so far poorly elucidated. Inhibition of Hsp90s or HSP70 has been put forward as a possible therapeutic intervention point for AD (Blair et al., 2014; Campanella et al., 2018). This increases HSF1 activity, which promotes transcription of many heat shock proteins and thus eventually leads to the expression of more chaperones (Campanella et al., 2018). These Hsps reduce the impact of cell stress levels, reduce Tau phosphorylation and Aβ aggregation, and maintain protein homeostasis (Campanella et al., 2018). The Hsp90 inhibitors can reduce Tau pathology in certain systems but have not been clinically tested yet (Blair et al., 2014).

Hsp90 inhibitors exist and have been tested in clinical trials for cancer therapy, which provides data on dosage, application and toxicity. If successful, such inhibitors will address multiple pathogenic events of AD development. Hsp90 inhibitors as drugs in neurodegeneration, however, have to overcome a few major challenges. 1) The fundamental challenge is that in contrast to cancer drugging does not aim at killing a cell, but instead keeping neurons alive. 2) Hsp90 chaperones have many important functions in the cell, and inhibiting Hsp90 chaperones may have pleiotropic effects. 3) The downside of most Hsp90 inhibitors is that most of them are not specific for a particular Hsp90 paralogue (Blair et al., 2014; Gewirth, 2016). Inhibitors targeting cytosolic Hsp90 paralogues may also inhibit TRAP1, due to the high degree of identity of key residues in the ATPase binding pocket (Gewirth, 2016). Together, while any strategy involving Hsp90 inhibition profits from many years of mechanistic, functional and clinical data, it faces also challenges that are not trivial to overcome. The fact that TRAP1 levels are strongly reduced in AD suggests that any Hsp90 inhibition strategy should spare TRAP1.

While AD is widely researched, no new treatment option has been approved since 2003 (Godyń et al., 2016). Current therapeutics in clinical trials focus on preventing the formation of NFTs, SPs and providing symptomatic relief (Godyń et al., 2016). More mechanistic insights are required to assess the relationship of TRAP1 to AD. TRAP1 may provide a link between the early pathological events of oxidative stress, glucose hypometabolism and mitochondrial dysfunction in disease. It will be rewarding to understand the exact prevalence of extramitochondrial TRAP1, its function and its export mechanisms are important to further elucidate. If TRAP1 is localised in multiple compartments, it might have a larger role in crosstalk between organelles. TRAP1 decrease will than have an even larger impact on cellular function than already determined.

Compounds modulating cellular protein quality control could provide a new approach on influencing AD pathology. Since the molecular cause of AD is unclear, it is imprudent to say TRAP1 up-regulation will cure the disease. However, if the cause of and mechanism behind TRAP1 down-regulation in AD is elucidated, we are one step closer to regain control over the derailed PQC. TRAP1, as the most reduced Hsp90, may thus provide new insights in the decades-old puzzle of AD development and onset.

## Abbreviations

β-HIVS, β-hydroxyisovalerylshikonin; Aβ, bèta amyloid; ANT1/2, adenine nulceotide translocator 1/2; APAF-1, apoptotic protease-activating factor 1; c-Src, proto-oncogene tyrosine-protein kinase Src; C/EBP, CCAAT enhancer binding protein; CTD, C-terminal domain; CypD, cyclophilin D; Cyt C, cytochrome C; DFO, deferoxamine; ER, endoplasmic reticulum; ETC, electron transport chain; GRP94, glucose-regulated protein 94; GSH, glutathione; GzmM, granzyme M; HSE, heat shock element; HSF1, heat shock factor 1; KO, knock-out; MD, middle domain; NAC, N-acetyl-cysteine; NTD, N-terminal domain; NXC, sodium-calcium exchanger; OXPHOS, oxidative phosphorylation; PARP, poly (ADP-ribose) polymerase-1; PHB2, prohibitin 2; PINK1, PTEN induced putative kinase 1; PQC, protein quality Control; PTP, permeability transition pore; RB, retinoblastoma protein; ROS, radical oxygen species; sHSPs, small heat shock proteins; SMAC, second mitochondria-derived activator of caspase; STAT3, signal transducer and activator of transcription 3; TBP7, tat-binding protein 7; TNFR1, type I tumor necrosis factor receptor; Tom70, translocase of outer membrane 70; TPR, tetratricopeptide; UPR, unfolded protein response; VDAC1, voltage-dependent anion channel 1.
